# Differences in Context and Feedback Result in Different Trajectories and Adaptation Strategies in Reaching

**DOI:** 10.1371/journal.pone.0004214

**Published:** 2009-01-16

**Authors:** Fritzie Arce, Itai Novick, Maayan Shahar, Yuval Link, Claude Ghez, Eilon Vaadia

**Affiliations:** 1 Department of Physiology, The Institute for Medical Research Israel-Canada, Hadassah Medical School, Jerusalem, Israel; 2 The Interdisciplinary Center for Neural Computation, Jerusalem, Israel; 3 Hebrew University, Jerusalem, Israel; 4 Center for Neurobiology and Behavior, Columbia University College of Physicians and Surgeons, New York, New York, United States of America; The University of Western Ontario, Canada

## Abstract

Computational models of motor control have often explained the straightness of horizontal planar reaching movements as a consequence of optimal control. Departure from rectilinearity is thus regarded as sub-optimal. Here we examine if subjects may instead select to make curved trajectories following adaptation to force fields and visuomotor rotations. Separate subjects adapted to force fields with or without visual feedback of their hand trajectory and were retested after 24 hours. Following adaptation, comparable accuracies were achieved in two ways: with visual feedback, adapted trajectories in force fields were straight whereas without it, they remained curved. The results suggest that trajectory shape is not always straight, but is also influenced by the calibration of available feedback signals for the state estimation required by the task. In a follow-up experiment, where additional subjects learned a visuomotor rotation immediately after force field, the trajectories learned in force fields (straight or curved) were transferred when directions of the perturbations were similar but not when directions were opposing. This demonstrates a strong bias by prior experience to keep using a recently acquired control policy that continues to produce successful performance inspite of differences in tasks and feedback conditions. On relearning of force fields on the second day, facilitation by intervening visuomotor rotations occurred only when required motor adjustments and calibration of feedback signals were similar in both tasks. These results suggest that both the available feedback signals and prior history of learning influence the choice and maintenance of control policy during adaptations.

## Introduction

While externally imposed perturbations initially degrade skilled reaching movements, humans learn to control their movements predictively rather than through successive corrections [1]–[5]. Computational models have suggested that endpoint variability and deviations from rectilinearity might be minimized concurrently based on a common performance error [6], [7]. Indeed, the characteristic bell-shaped velocity profiles and rectilinear trajectories of horizontal reaching movements [8]–[10] were simulated robustly and with varied dynamic loads, through minimization of endpoint variability alone [11]. Recent models propose an alternative view suggesting that to maximize performance, trajectories may be “reoptimized” and have a curved shape [12]. Indeed, various types of curved trajectories were reported; for example, when subjects reached along a curved path that conformed to the stiffness of a virtual disk [13], or when visual feedback was displayed in joint rather than in Cartesian coordinates [14], or when adapting to visuomotor rotations when visual feedback was limited to the endpoint [15]. Scheidt and Ghez (2007) suggested that their results were accounted for by a computational model in which intended trajectories and final hand position are driven by separate controllers.

Intrigued by these studies, we reasoned that while the spatio-temporal criteria for success constitute explicit demands of the task, trajectory shape is not uniquely determined by this constraint but is influenced by other factors such as calibration of the available feedback signals for the state estimation required by the task. For example, when reaching to an object, estimating the hand position is not as certain when we cannot see the hand as when we can see it [16], [17]. This uncertainty can lead to errors in sensory estimates and consequently to movement variability [18]. Estimation of the state of the body and the environment is all the more critical when reaching in novel environments. Here we examine how differential visuospatial information provided at movement termination and during movement itself influences adaptive adjustments to externally imposed force field perturbations during reaching. We hypothesized that while accuracy might be recovered with terminal visual feedback alone, rectilinearity might require continuous visual feedback for proprioception to be calibrated over the entire workspace [19]. Thus, in the absence of vision, the shape of adapted trajectories would be constrained by the predicted effects of force fields afforded by the available feedback.

In a first set of experiments, we compared movements made by two groups of subjects reaching for a single target that changed color when acquired successfully. One group received only this feedback (i.e. target color change) while the other could also see a cursor during the entire movement. Recovery of both terminal accuracy and straight hand trajectories in both groups would support the idea that both are driven by the same motor errors, as suggested by optimal control models. Instead, rectilinear trajectories were recovered only when subjects had continuous visual feedback of their hand trajectory, but remained curved when feedback was limited to success at movement termination. This was consistent with different estimates of the expected trajectories in the two conditions and suggested that proprioceptive and visual feedback operated differently in regulating trajectory shape.

In a second set of experiments, we examined how the control strategies learned during force field adaptation influenced subsequent adaptation to visuomotor rotations, where movements were under continuous visual control. Optimal control would predict rectilinear trajectories in both cases. Instead, prior experience biased adapted trajectory shapes (straight vs. curved) in rotation learning. In these same subjects, we then examined the effects of intervening visuomotor rotation. If the control strategies used in the intervening rotations were similar, the effects on force field relearning would be similar regardless of feedback conditions and depend only on the direction of the imposed rotation that had been learned. This was not found. Rather, there were distinct effects on trajectories depending on the direction of the imposed rotation and on feedback conditions. The findings have been reported previously in abstract form (Arce et al., *Soc. Neurosci. Abstr.* **413.14**, 2007).

## Materials and Methods

### Ethics Statement

The experimental procedures were approved by the Hebrew University institutional review board. All subjects gave informed written consent prior to the experiment.

### Subjects

Thirty-eight subjects (age range: 19–34) were paid for their participation. All subjects were naïve to the experimental goals and reported having normal or corrected vision, absence of neurological deficits, right-handedness, and scored above 75% in a hand-dominance questionnaire.

#### Procedure

We conducted two experiments; subjects in Experiment 1 (two groups, 6 subjects in each) adapted to a force field either with or without visual feedback (VFB). Another set of subjects in Experiment 2 (4 groups, 5 subjects in each) were exposed to two perturbations sequentially: force field (either with or without VFB) followed by visuomotor rotation of a similar or opposite direction to force field. As a control group for experiment 2, another set of subjects (n = 6) adapted to a single visuomotor rotation. Each subject was randomly assigned to only one group (Table 1).

**Table 1:** Experimental groups and block structure.

	Day1	Day2	n
**Experiment 1: Single perturbation groups**			
Force field - vision (FFv)	S, FFv, S	S, FFv, S	6
Force field - no vision (FFnv)	S, FFnv, S	S, FFnv, S	6
**Experiment 2: Double perturbation groups**			
Matched FFv-rotation (FFvR−)	S, FFv, R−, S	S, FFv, S	5
Matched FFnv-rotation (FFnvR−)	S, FFnv, R−, S	S, FFnv, S	5
Non-matched FFv-rotation (FFvR+)	S, FFv, R+, S	S, FFv, S	5
Non-matched FFnv-rotation (FFnvR+)	S, FFnv, R+, S	S, FFnv, S	5
Control Rotation (R−)	S, R−, S	S, R−, S	6

**Figure 1:**
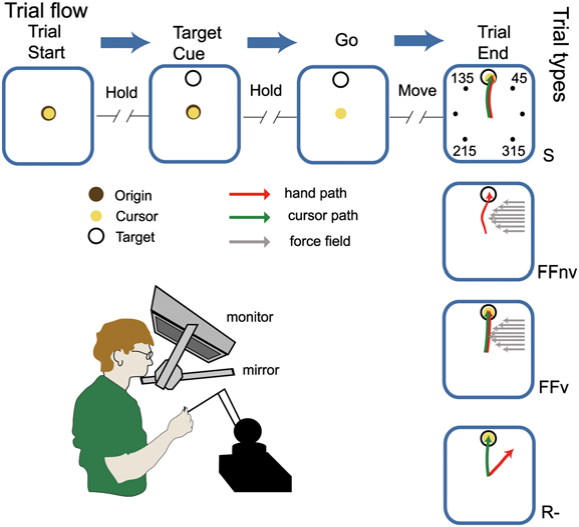
Experimental set-up and paradigm. Subject, with the head placed on a chinrest and the arm in a natural posture, reaches to a target projected onto a mirror using a robotic arm. Vision of the subject's hand and robotic arm is occluded. Trial flow (horizontal) - the sequence of events in a trial that could be one of 4 types (vertical): standard (S), force field without visual feedback (FFnv), force field with visual feedback (FFv), or visuomotor rotation (R−). Perturbations are only introduced in blocks with a single target and never in standard trials. In FFnv, the subject does not see the cursor during the reach but does in FFv (yellow circle and green arrow). In R−, the hand to cursor mapping is rotated 45° counterclockwise such that the subject has to move the hand to 45° in order to bring the cursor to a 90°-target.

Subjects sat in front of a workstation, grasping the handle of a lightweight robotic arm (Phantom Premium 1.5 High Force, SensAble Devices, Cambridge, MA), with their chin on a chinrest (Fig. 1). They adopted a natural arm posture, their upper arm in a near-vertical plane, their hand at about shoulder level and were to move the robotic arm from a common starting location to one of several peripheral targets. A 3D monitor projected onto a mirror a stereo image of spherical targets and a cursor that tracked the instantaneous position of the robotic arm's handle. Subjects did not see their hand or the robotic arm while performing the task. Reaching movements were constrained to the horizontal plane (created via force boundaries applied by the robotic arm along the vertical axis) and involved rotations of shoulder, elbow, and wrist joints.

### Experiment 1: Single force field perturbations

#### Trial events

Figure 1 describes the sequence of events during different trial types. Trials started with the appearance of a sphere (12 mm-radius) in the center of the virtual workspace that served as an *origin* and a cursor (sphere of 9 mm-radius) indicating the current position of the robotic manipulator end-point. Subjects were instructed to position the cursor at the origin and to hold this position for a random period (0.1–0.5 s) after which a peripheral target (12 mm-radius) appeared. After another random period (0.1–0.5 s) from target appearance, the origin disappeared. This served as a go-signal. Subjects were instructed to respond by reaching for the target as accurately and as fast as possible. The trial was terminated 1 s after the go-signal. Movements were successful if the hand reached the target within this time period and were cued by target color change and an auditory cue (notify.wav, Microsoft Windows). Trials were aborted when subjects did not respond within 1 s, moved prior to the go-signal, or moved in the wrong direction (exceeding a perpendicular deviation of 18 mm from a line connecting the centers of the origin and target). In these circumstances, subjects were presented with another auditory cue (Pop-up Blocked.wav, Microsoft Windows). At trial end, force field if present (see below), was turned off and the workspace was blanked. An intertrial interval (0.5–1 s) immediately followed trial termination.

#### Block and trial types

All subjects participated in two day-sessions separated by 24 hours. Each day session started and ended with a standard block. In the *standard block* (176 trials), subjects reached to eight radially located targets (separated by 45°, at 70.71 mm from a common origin) presented in random order. The cursor was displayed continuously from start to end of trial. Hand and cursor movements were overlaid. The *perturbation block* followed the first *standard block* after a rest period of randomized duration (45–60 s) provided between blocks. Unlike the 8 targets in the standard block, subjects reached to a **single target at 90°** in the presence of a viscous curl force field either with or without visual feedback of the cursor position (220 trials). In force field without visual feedback, the cursor disappeared at go-signal and reappeared only at the start of the next trial when the cursor approached the vicinity of the origin (20 mm-radius). The force field was applied to the hand only during reaching and always pushed the arm perpendicular to its current velocity in counterclockwise direction (indicated as negative). It was generated using the following equation:
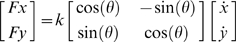
where *Fx* and *Fy* are robot-generated forces, *k* = 6 Ns/mm, θ = −90°, 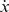 and 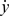 are the components of the hand velocities in the horizontal plane. As subjects held their arm and forearm approximately in a vertical plane (Fig. 1), the effects of the force fields were distributed throughout the limb. However, since the hand was maintained in the horizontal plane by the robotic arm, perturbations moving it to the left were mainly associated with internal rotation and adduction of the shoulder. The force perturbation was engaged when the target appeared and deactivated upon trial termination. Thus, with a single adaptation target, subjects experienced the perturbation only while moving in a narrow range of directions and not when returning the hand to the origin.

To assess retention and the effect of additional practice, subjects returned 24 hours later and were given the same set of blocks as in the previous day. At the completion of the experiment, subjects were asked to describe in writing the tasks they had been given and the strategies they used.

### Experiment 2: Double perturbations

Four new groups of subjects adapted to visuomotor rotations immediately after the force field block performed either with or without VFB (Table 1). Visuomotor rotations consisted of a 45°-rotation of the cursor location relative to the hand position, using the following equation:
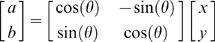
where *a* and *b* are the coordinates of the “rotated” hand position, θ = ±45°, *x* and *y* are the components of the hand position in the horizontal plane. While the prior force field perturbation was always counterclockwise, the direction of the visuomotor rotation was either “matched” (counterclockwise) or “non-matched” (clockwise). The target direction (90°) was the same for the two perturbations although the final positions of the required movement were different (i.e. 135° for non-matched and 45° for matched rotation).

Subjects always started with a standard block, followed by force field, then rotation, and ended with another standard block. To test retention of force field learning, subjects returned on the second day to do a block of the same force field learned previously. Standard blocks preceded and followed the force field block. Trial events were as described in Experiment 1.

#### Data analysis

Hand position was sampled at 100 Hz by the device encoders and low-pass filtered (cut-off frequency 20 Hz) using Matlab filter toolbox (The Mathworks, Inc., Natick, MA) prior to computing hand velocities. Movement onset was marked when hand velocity last exceeded a threshold of 0.02 m/s prior to reaching two-thirds of peak velocity. All trials, whether successful or not, were included in the analysis except for *aborted trials* when subjects did not respond within 1 s from go-signal, or initiated the movement before the go-signal, or when hand velocity remained under 0.08 m/s.

We evaluated task performance itself by assessing success rate and the accuracy at movement endpoint, when subjects were informed of the success or failure of a given movement. Trial-by-trial changes in accuracy were measured by the perpendicular distance between the target's center and the final position reached at the end of the trial, and termed *spatial error*. Changes in systematic and variable error were computed using principal components analysis, from the centroids and areas of the 95% confidence ellipses of the endpoint distributions (first and last 40 trials of each day's perturbation blocks).

To evaluate hand trajectories, we computed the initial directional deviations and the path curvatures. *Directional deviation* was taken as the angular difference between the direction of a vector going from the hand position at movement onset to the target and one from the origin to the hand position 150 ms after movement onset. This early time-point excludes visual feedback effects [20], [21]. *Path curvature was* taken as the mean perpendicular distances of individual points along the path to a straight line connecting the origin to the target (from movement onset to trial termination).

We examined the effects of practice on performance. Differences in adaptive changes between feedback conditions and across early (first 20 trials) and late phases (last 20 trials) of training were assessed. We used a mixed model ANOVA with feedback condition and learning phase as fixed effects, subjects as random effects and nested into the group variable. When interactions between feedback conditions and phase were found non-significant, ANOVA was run again to exclude the interaction term. When effects were found significant, a separate mixed model ANOVA was performed if applicable. *Post-hoc* paired comparisons were performed with the Tukey-Kramer correction. To compare retention on the next day in the double perturbation groups (Experiment 2), we calculated an *improvement index* (*IMP*) as a normalized trial-by-trial difference between group mean values obtained on the first 40 trials of day1 and day2 for each learning variable (*IMP*(*i*) = (error1(*i*)−error2(*i*))/(error1(*i*)+error2(*i*)). Differences in *IMPs* were tested using one-way ANOVA. *Post-hoc* paired comparisons were performed with the Tukey-Kramer correction. Significance level for all tests was set at *p* = 0.05.

## Results

### Experiment 1. Adaptation to force fields: differential effects of visual feedback on endpoint and trajectory

#### Terminal accuracy

**Figure 2:**
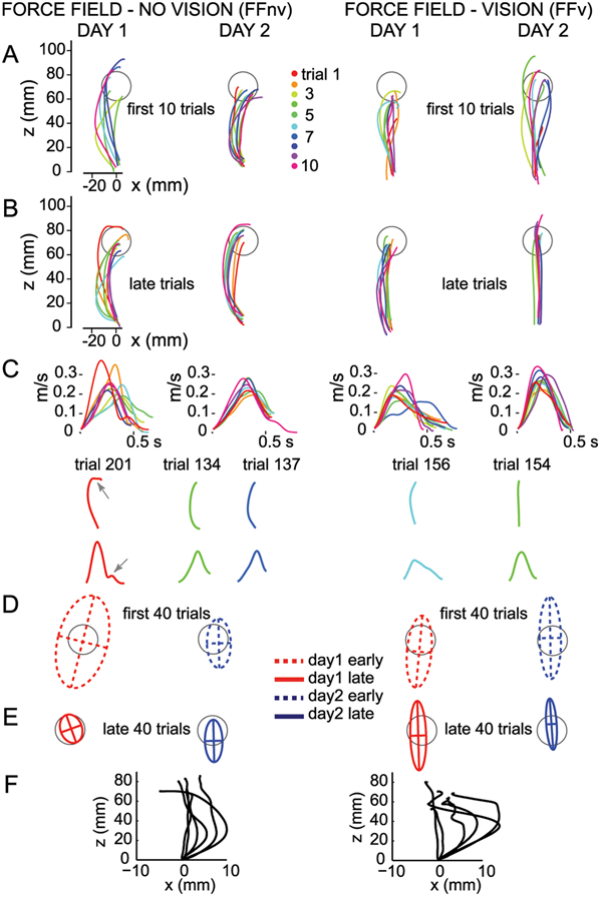
Adaptation to force fields with and without visual feedback: Single subjects. A–B, Day1 and day2 hand paths of two single subjects during the first 10 trials and late trials (ranging from trial 131–220) of force field without visual feedback (FFnv, left) and with (FFv, right). Aborted trials (see methods) are not shown. Hand paths, plotted from detected movement onset to movement end, show displacement from origin to a target at 90° (gray circle). C, Velocity profiles of the hand paths in the late trials shown in B. Representative single-trial hand paths and their corresponding velocity profiles are also shown. In FFnv, the smooth early phase of the velocity profiles showed that in most cases, the path curvature in the late trials was not due to online corrections. However, in some cases trajectory corrections were observed as reflected in the presence of inflections after peak velocity (gray arrows, trial 201). D–E, Endpoint variability. Shown are 95% confidence ellipses (per subject) for early and late trials of both days. Gray circle shows the size of the target for comparison. F, Aftereffects. Hand paths of subjects in FFnv (n = 6) and FFv (n = 6) corresponding to the first trial in the learned direction (90°) in the post-learning standard block. Starting points of hand paths were aligned at (0,0) for easy comparison of directional deviations. Hand paths were deviated in the direction opposite to that of force field. Since this first trial could occur after several trials in this block, the aftereffect could be smaller. For this reason, aftereffects are used here for illustration only.

**Figure 3:**
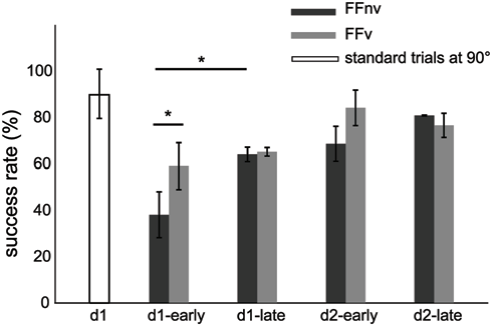
Success rates. Success rates in early and late adaptations to force fields with (FFv) and without visual feedback (FFnv) on day1 and day2. Success rate was calculated in 10 bins of 22 trials each. Each bar depicts the mean of the first or last 3 bins across all subjects in the group. The mean success rate for standard trials for the same direction (n = 22) is also shown for FFnv. Vertical line is ±1 standard deviation (* = p<0.05).

When first experienced, force fields deviated the subjects' hand perpendicular to the direction of movement (Fig. 2A, day1) and success rate decreased dramatically (Fig. 3). This occurred in both force field conditions but the effect was greater without VFB than with VFB (Fig. 3, d1-early *F*_(1,10)_ = 6.0 *p* = 0.03) consistent with feedback correction of initial errors. With practice, success rates increased as movements became more accurate (Fig. 2A vs. 2B) and precise (Fig. 2D vs. 2E). By the end of the first training day, success rates became similar with and without VFB (d1-late: *F*_(1,10)_ = 0.3 *p*>0.10) and improved further on the second day to the same degree (Fig. 3, day2 *p*>0.10).

**Figure 4:**
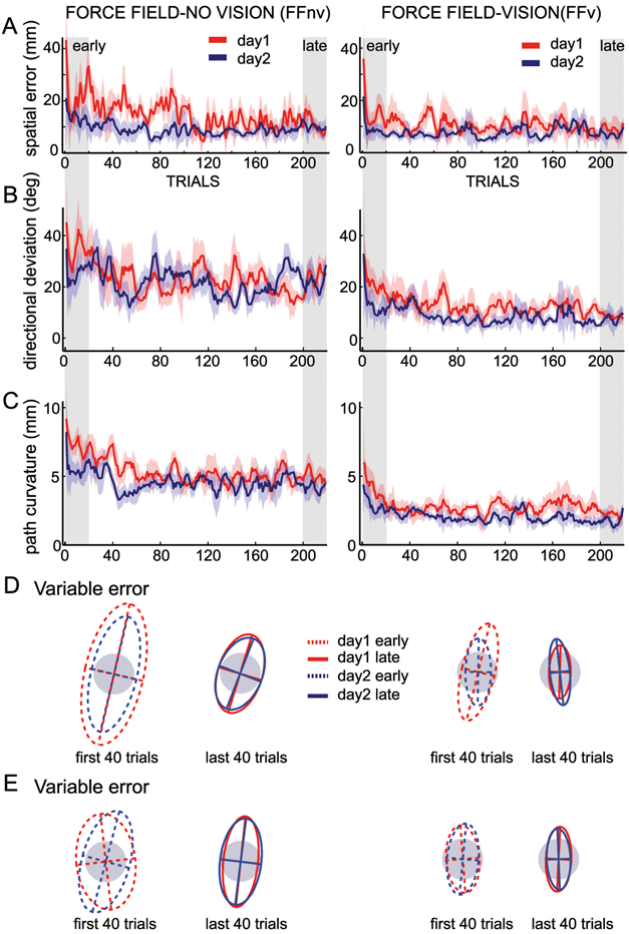
Adaptation to force fields with and without visual feedback. Group data, A–C, Day1 and day2 time courses, showing trial-by-trial means and ±1 SEM of spatial errors, directional deviation, and path curvature respectively, for force field without visual feedback (left) and with visual feedback (right). Shaded areas correspond to early and late trials used for comparisons. The directional deviation of the first trial in FFnv was the mean across 3 subjects only (the other 3 were aborted trials). D, Endpoint variability ellipses for early and late trials for each group. Each subject's endpoint position for each trial was subtracted from his mean endpoint position. Gray circle shows the size of the target for comparison. E, As in D but using endpoints taken at near zero velocity.

Figure 4 (A,D–E) shows the improvements in spatial accuracy and precision from early to late trials with and without VFB. Comparable endpoint accuracies were achieved with and without VFB (ANOVA, main effect of group: *F*_(1,10)_ = 1.4, *p*>0.10, see also figure S1 for the mean endpoints of each subject in both groups). Note that early in adaptation (see Fig. 4A-early, compare shaded red traces in FFnv vs. FFv), spatial errors seemed higher without VFB than with VFB but this difference did not reach significance level. For both groups, spatial errors showed significant reductions across training days (main effect of phase: *F*_(3,10)_ = 27.8, *p*<0.00001). To compare between the different learning phases, follow-up ANOVAs (mixed model, fixed effect of phase and random effect of subjects) for each group were done. Subjects in both groups reduced their spatial errors substantially from early to late trials on day1 (Fig. 4A compare red traces, FFv: *F*_(3,5)_ = 9.6, *p*<0.00001, FFnv: *F*_(3,5)_ = 20.3, *p*<0.00001, *post-hoc p*<0.001). On day2, savings were apparent for both groups; spatial errors were significantly lower in the early trials of day2 than day1 (Fig. 4A-early, compare red vs. blue traces, *post-hoc p*<0.01). Accuracy did not improve further from early to late trials on day2 (Fig. 4A blue traces, *post-hoc p*>0.10 for both groups).

Mean directional deviations at movement termination (i.e. the angular difference between the direction of a vector going from the hand position at movement onset to the target and one from the origin to the hand position at trial termination) were also similar with and without feedback. While there was a small difference between groups (ANOVA, main effect of group: *F*_(1,10)_ = 5.8, *p* = 0.04), this was only present at the end of the first training day (FFnv: M = 5.0° SEM = 5.5, FFv: M = 2.3° SEM = 1.9, *F*_(1,10)_ = 6.1, *p* = 0.03) and beginning of day2 (FFnv: M = 7.5° SEM = 8.9, FFv: M = 3.1° SEM = 2.9, *F*_(1,10)_ = 6.1, *p* = 0.03). By the end of day2, subjects achieved similar directional deviation at movement termination with and without VFB (FFnv: M = 4.4° SEM = 5.5, FFv: M = 2.5° SEM = 4.3, *F*_(1,10)_ = 2.5, *p*>0.10).

The variability in endpoint distributions (i.e. precision), measured as the areas of the 95% confidence ellipses, was also reduced with practice in both groups (Fig. 4D–E day1 red dotted vs. solid line, main effect of phase: *F*_(3,10)_ = 8.1, *p* = 0.0004). Like spatial error, reductions were significant from early to late trials on day1 (follow-up mixed ANOVA, FFv: *F*_(3,5)_ = 3.6, *p* = 0.04, *post-hoc p*<0.05; FFnv: *F*_(3,5)_ = 6.4, *p* = 0.005, *post-hoc p*<0.01); No further significant reductions were observed on day2 (*p*>0.10). Precision was greater with vision than without vision early on day1 (Fig. 4E dotted traces, day1, main effect of group: *F*_(1,10)_ = 5.5, *p* = 0.04, follow-up one-way ANOVA, *p*<0.01) but no longer at the end of training (*p*>0.10 for all other phases). The endpoint distributions used in figure 4E were measured when subject received feedback of trial-end. To verify that these improvements in precision did not reflect the truncation of ongoing movements by trial termination, we also performed the same set of analyses on final hand positions at velocity minima (which could occur after the trial termination). Similar results of improved precision with adaptation were obtained for the two measures of movement endpoint as shown by comparing figures 4E and 4F (for all comparisons *p*>0.10).

#### Trajectories

Although subjects achieved comparable success levels and accuracies with and without VFB, movement trajectories differed systematically. Directional deviations early in the movement were substantially larger and hand paths consistently more curved without VFB than with VFB (Fig. 4B–C, group effect for directional deviation: *F*_(1,10)_ = 8.6 *p* = 0.01 and for curvature: *F*_(1,10)_ = 15.8 *p* = 0.003). Importantly, trajectories remained different for the two groups even after a second day of training and the same degree of accuracy (Fig. 4B–C blue traces, follow-up mixed ANOVA, directional deviation: *F*_(1,10)_ = 5.5 *p* = 0.04, curvature: *F*_(1,10)_ = 8.7 *p* = 0.01). Path curvatures were significantly reduced in both groups (phase effect: *F*_(3,10)_ = 51.9 *p*<0.00001). It should be noted that without VFB, path curvature stabilized at a new value which remained similar on day2, even as accuracy improved (follow-up mixed ANOVA, *F*_(3,5)_ = 25.3 *p*<0.00001, *post-hoc p*>0.10). With VFB, curvatures were further reduced from early to late trials of day2 (*F*_(3,5)_ = 28.7 *p*<0.00001, *post-hoc p*<0.001). Since endpoint errors were similar at the end of practice across feedback conditions, this suggests that subjects learned to move their hand through different planned trajectories.

Nevertheless, adaptive changes in initial trajectory, although to different degrees, were present in both groups. First, the directional deviations produced by the perturbations were reduced progressively with practice both with and without VFB (Fig. 4B–C, phase effect: *F*_(3,10)_ = 35.1 *p*<0.00001). This variable was measured 150 ms after movement onset, before any corrective adjustments would be possible. Thus, changes in feedforward commands reduced the effect of the perturbing forces. Second, *aftereffects* occurred when the force field was removed (Fig. 2F). Third, savings on day2 were also apparent in the significantly lower directional deviations and curvature on the early trials of day2 than day1 (Fig. 4B–C red vs. blue traces, *post-hoc p*<0.001).

Subjects might have improved terminal accuracy without visual feedback by increasing movement time and perhaps using this added time for corrective movements [22]. However, following adaptation, movement durations of reaches were similar with and without VFB (late day1: FFv: M = 590 ms, SEM = 130; FFnv: M = 619 ms, SEM = 18, *F*_(1,10)_ = 0.11, *p*>0.10; late day2: FFv: M = 623 ms, SEM = 125; FFnv: M = 610 ms, SEM = 134, *F*_(1,10)_ = 0.47, *p*>0.10). Velocity profiles and their peak values were also similar with and without VFB (day1, FFv:M = 0.2 m/s SEM = 0.08; FFnv: M = 0.19 m/s SEM = 0.07; ANOVA, *F*_(1,10)_ = 0.11, *p*>0.10; day2, FFv:M = 0.17 m/s SEM = 0.06; FFnv:M = 0.19 m/s SEM = 0.06, *F*_(1,10)_ = 1.0, *p*>0.10). Furthermore, as can be seen in the sample profiles of figure 2C, inflections (sub-movements) occurred mainly at the end of movement and generally disappeared over the course of practice on both days. Thus, the persistent curvature did not reflect corrective sub-movements but represented a change in the feedforward control of the desired trajectory in the presence of force field.

Note that subjects did not feel any force field in the return movement because the field was disengaged at trial termination (see Methods, Block and trial types). This, however, did not constitute a washout effect between trials. Performance errors were substantially reduced within the first 10 trials in the with VFB condition (Fig. 4). If washout had been significant, learning rates would be slower. Also, since generalization is confined to narrow angular disparities from the learned direction [23]–[25], there is little concern about the return movements to 180°, opposite from the learned direction.

In sum, learned improvements in endpoint accuracy were achieved differently in the two feedback conditions: hand trajectories remained curved when subjects could not see the cursor but rapidly became straight when VFB was available. These findings agree with the notion of separate processes governing endpoint and trajectory [15].

### Experiment 2. Interactions between force field and rotation learning

#### Differential effects of learned force fields on subsequent adaptation to rotation

**Figure 5:**
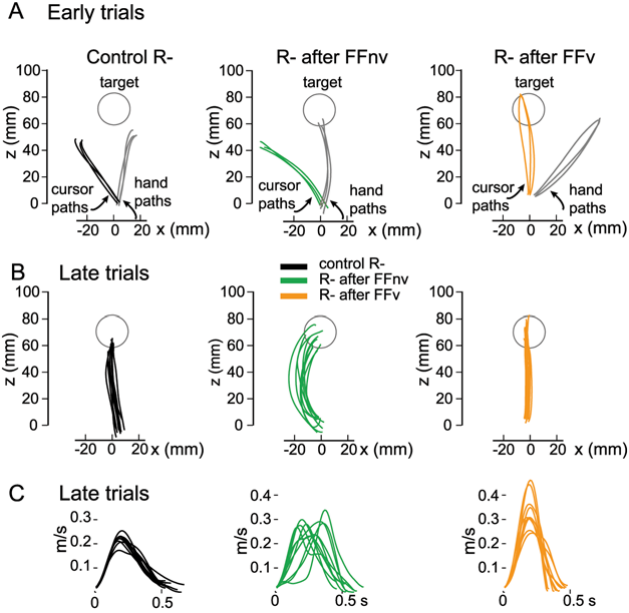
Double perturbations of matched directions: Visuomotor rotation adaptations after force field adaptations. A, Cursor and hand paths of representative subjects (one per group) during the first trial of the first exposure to rotation (*left*, Control R−), and following adaptations to force field without visual feedback (*center*, R− after FFnv) and with (*right*, R− after FFv). To reach a target at 90° in visuomotor rotation, subjects should direct their movements 45° clockwise from the target. Subjects see a rotated cursor feedback of their hand movement and final hand position such that they see the cursor reaching 90°-target while their hands end at 45°. *Center and right*, the hand path of the first rotation trial (solid gray line) are also shown to illustrate aftereffects of prior force field adaptations. B, As in A, cursor paths of the late rotation trials. The hand paths of the subject who had prior adaptation to FFnv were curved (center) as opposed to the straight paths of the subjects in control R− and in R− after FFv. C, Velocity profiles of the late trials shown in B. The profiles of the subject in R− after FFv show that the curved paths were not due to online trajectory corrections, as seen previously in single force fields without VFB.

We next examined whether adaptation to force fields—with and without visual feedback—differentially influenced movements made during later adaptation to visuomotor rotations, where visual feedback was now used to reach the target. New groups of subjects were exposed first to force fields (either with or without VFB) and next to rotations (Table 1, Experiment 2). These groups adapted to rotations that were either matched to the force field direction (counterclockwise) or non-matched (clockwise). With direction-matched rotation, the rotated visual cursor was deviated in the same direction as the initial movement when encountering the force perturbation (Fig. 5A-left, cursor paths). For non-matched rotations, the directions of the rotation and force perturbations were opposite. Note however that the actual final hand positions at target acquisition in the two tasks were different (i.e. 90° for force fields and 45°/135° for matched/non-matched rotations). We expected that if the motor adjustments compensating for the initial force field perturbation and those needed for the subsequent adaptation to rotation were in the same direction (as in matched directions), adaptation would be facilitated. If they were opposite (non-matched directions), there would be interference. If prior history were not taken into account, adaptations to rotation would yield straight trajectories and accurate endpoints whether subjects previously learned force fields with or without feedback.

As in Experiment 1, the trajectories of subjects in the double perturbation groups were curved when adapting to force fields without VFB and straight when VFB was present. However, the trajectories developed during visuomotor rotations differed between these conditions (Fig. 5B, orange vs. green).

#### Matched directions

**Figure 6:**
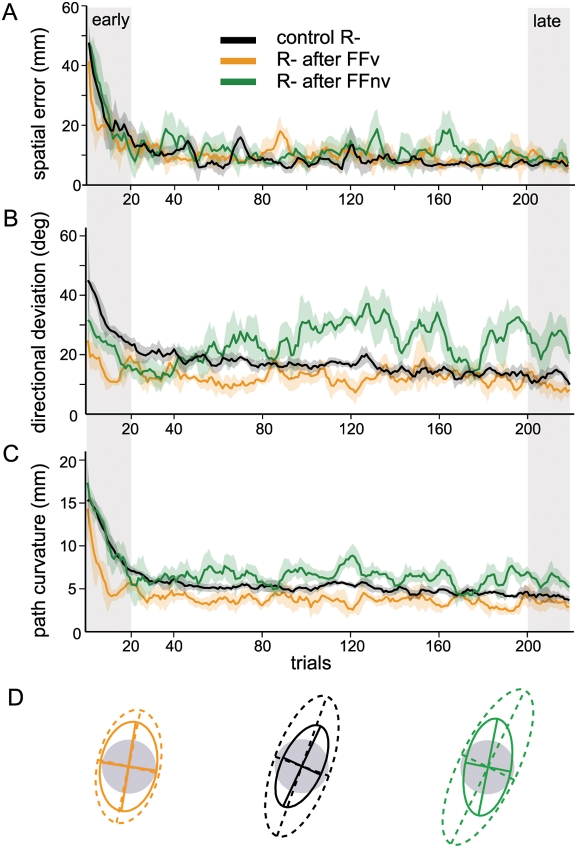
Effects of prior force field adaptations on matched visuomotor rotations. Group data. A–C, Time courses for the different movement parameters during adaptations to single rotation only (control R^−^) and to rotations after learning force field with visual feedback (R− after FFv) and without (R− after FFnv). D, Endpoint variability ellipses of the first 40 (dotted lines) and last 40 (solid lines) rotation trials for all subjects in each group.

With visuomotor rotations alone, subjects compensated for the perturbation with successful target acquisition and accuracy by the 10^th^ trial onwards. The time course of the reduction in spatial errors was similar in subjects that had previously adapted to force fields, both with and without VFB as in rotation (Fig. 6A, group effect: *F*_(2,13)_ = 0.8, *p*>0.10, phase effect: *F*_(1,13)_ = 195.0, *p*<0.00001). Endpoint variability was also reduced substantially (Fig. 6D, phase effect *F*_(1,13)_ = 10.8, *p* = 0.005) and to the same degree in all (group effect *F*_(2,13)_ = 0.6, *p*>0.10).

On the other hand, there were significant effects of the previous force field adaptation on the initial deviations (group effect *F*_(2,13)_ = 5.9, *p* = 0.02; phase effect *F*_(1,13)_ = 5.2, *p* = 0.04; interaction effect *F* = 6.1, *p* = 0.01) and shapes of adapted trajectories (group effect *F*_(2,13)_ = 4.2, *p* = 0.04; phase effect *F*_(1,13)_ = 126.4, *p*<0.00001) in visuomotor rotations. Hand movements made during the first rotation trials after adapting to force fields were initially directed clockwise in comparison to movements made with the control rotation (Fig. 5A, hand paths). This is likely to be an aftereffect of force field adaptation since there were no washout trials between the two perturbations. The relative clockwise deviation was close to the direction required to compensate for the cursor rotation by moving the hand 45° clockwise. As in single force field perturbations (Experiment 1), the aftereffects on rotation were larger when force field had been learned with VFB than without VFB. Indeed, early directional deviations in the group that had prior VFB were significantly lower than those found in control rotation (Fig. 6B-early compare orange to black, follow-up mixed ANOVA *F*_(1,9)_ = 6.8, *p* = 0.03). In contrast, subjects who had previously adapted without VFB were not significantly different from control rotation subjects (Fig. 6B-early compare green to black, *F*_(1,9)_ = 0.5, *p*>0.10). Path curvature was comparable across all groups early in adaptation (Fig. 6C-early, p>0.10 for all comparisons).

As adaptation progressed, the trajectory shapes of the groups diverged. Trajectories rapidly became straight both with control rotations and after adapting to force fields with VFB but became curved when they had adapted to force fields without VFB (Fig. 5B–C, orange vs. green). While directional deviations with only rotation and with rotation after force field with VFB were similar (Fig. 6B-late, follow-up mixed ANOVA *F*_(1,9)_ = 0.1, *p*>0.10), mean directional deviation was significantly larger when rotation was experienced after force field without VFB from around the 40^th^ trial onwards compared to control rotation (*F*_(1,9)_ = 11.3, *p* = 0.008) and to rotation after force field with VFB (*F*_(1,9)_ = 9.2, *p* = 0.02). Curvature without VFB was also higher relative to control rotation (Fig. 6C-late, *F*_(1,9)_ = 9.5, *p* = 0.01) and rotation after force field with VFB (*F*_(1,9)_ = 8.2, *p* = 0.02). Note that the late-trial curvatures were in the direction opposite to the early-trial curvatures (compare figure 5A-center vs. 5B-center).

The re-emergence of curvature with rotation learning after force field without VFB suggests that subjects applied the same trajectory control strategy to achieve accurate termination in one context (force field no VFB) to another (rotation with VFB). This implies that the cost of switching strategies may be higher than maintaining a recently acquired curved trajectory plan, which allowed subjects to achieve success in a different task.

#### Non-matched directions

When perturbation directions were non-matched, prior adaptation to force fields did not have demonstrable effects on spatial accuracy and variability, as found in matched directions. Spatial errors in opposite rotations after force fields with and without VFB were reduced (phase effect *F*_(1,13)_ = 243.4, *p*<0.00001) but were not significantly different from each other nor from control rotation (group effect *F*_(2,13)_ = 1.6, *p*>0.10). Ellipse areas were also reduced but were similar across groups (group effect: *F*_(2,13)_ = 2.5, *p*>0.10; phase effect: *F*_(1,13)_ = 16.7, *p* = 0.001).

Like in matched-directions, significant effects of prior force field were found on the directional deviations during adaptation to non-matched rotation (group effect *F*_(2,13)_ = 6.9, *p* = 0.009; phase effect *F*_(1,13)_ = 230.0, *p*<0.00001). With the non-matched rotations, aftereffects of force fields would be in the direction opposite to the movement required to adapt to the rotation. Correspondingly, directional deviations were larger after force field with VFB than in control rotation (follow-up mixed ANOVA *F*_(1,9)_ = 13.6, *p* = 0.005). This interference early in adaptation diminished over successive trials and ceased at the end of the training block (*F*_(1,9)_ = 3.8, *p*>0.10). In non-matched rotation after force field without VFB, significant interference emerged after the first 20 trials and continued till late in adaptation (*F*_(1,9)_ = 6.3, *p* = 0.03). Path curvature showed significant reductions but were similar in all groups (phase effect: *F*_(1,13)_ = 276.2, *p*<0.00001; group effect: *F*_(2,13)_ = 1.0, *p*>0.10). Thus, adapted trajectories in subsequent opposite rotation were rectilinear inspite of previously acquired curved trajectories in force field without VFB.

To summarize the results of this section, we show that prior force field adaptations influenced adapted trajectory but not accuracy during later adaptation to visuomotor rotations. The effects of prior force field on the adapted trajectories in subsequent rotations were apparent regardless of differences in task demands and feedback conditions. When perturbation directions were matched, previously learned trajectories in force fields with and without VFB were carried over to subsequent rotation adaptation. When perturbation directions were non-matched, interferences in directional deviations occurred and precluded transfer of learned trajectory shapes.

### Differential effects of rotation adaptation on force field relearning

Here we examine how adaptation to visuomotor rotation influenced relearning of force fields (with and without VFB) the next day. To isolate these effects from effects on retrieval [26], [27], subjects performed two blocks of standard reaches, i.e. after the rotation block on day1 and before the force field block on day2. Relearning was assessed using indices of improvement from early trials of day1 to day2 (*IMPs*, see Data analysis). If the control strategies used in the intervening rotations were similar, the effects on force field relearning would be similar regardless of feedback conditions and depend only on the direction of the imposed rotation that had been learned.

**Figure 7:**
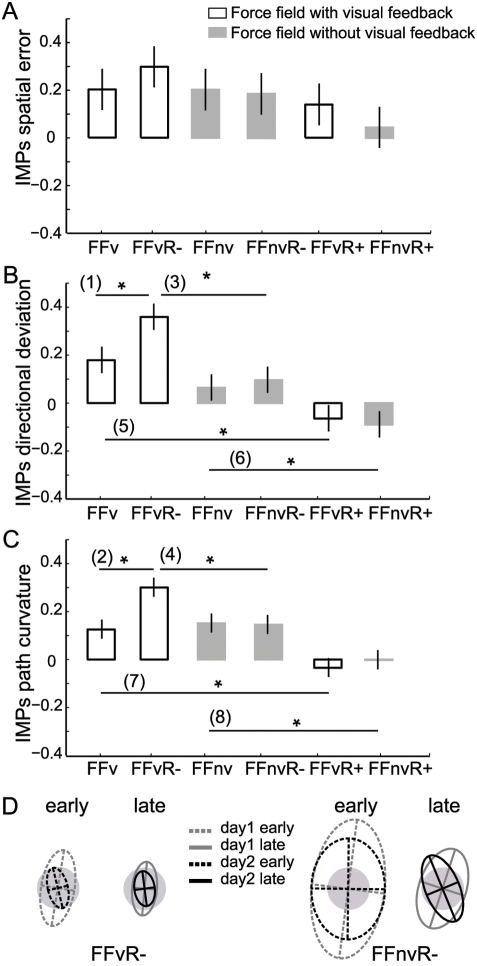
Effects of visuomotor rotation on force field retention. A–C, Improvement indices (IMPs) show increments in adaptations to force field from day1 to day2. Shown are mean IMPs of the first 40 trials for all groups. Vertical lines are 95% confidence interval of between-group differences in means. Numbered horizontal bars with asterisk indicate significant differences between paired groups (p<0.05). D, Endpoint variability ellipses of all subjects in the matched double perturbation groups.

Rotation adaptation did not affect endpoint accuracy and variability when relearning force field 24 hours later whether or not visual feedback was available and whether perturbations were matched or non-matched. *IMPs* in spatial error of all double perturbation groups were not significantly different from their respective control single force fields (Fig. 7A compare FFv vs. FFvR^−^/ FFvR^+^ and FFnv vs. FFnvR^−^/ FFnvR^+^, *F*_(5,234)_ = 3.8, *p* = 0.002; *post-hoc p*>0.10). The same was true for *IMPs* of endpoint variability (*F*_(5,26)_ = 0.5 *p*>0.05).

In contrast, the effects of intervening visuomotor rotation on the trajectories during force field relearning depended on the perturbation directions and feedback condition (Fig. 7B–C). *IMPs* were highest in matched directions with VFB (FFvR^−^) and lowest in non-matched directions (FFvR^+^ and FFnvR^+^). When perturbations were direction-matched, adaptive changes in directional deviation were facilitated in force field with VFB (group FFvR^−^, *F*_(5,234)_ = 17.8 *p*<0.00001) as seen in the significantly greater *IMPs* compared to control FFv (Fig. 7B comparison 1, *p* = 0.025). In contrast, adaptive adjustments produced by intervening rotations had no effect when re-experiencing force field without VFB (group FFnvR^−^, *p*>0.10). Correspondingly, improvements in the direction-matched groups were significantly higher with vision than without (Fig. 7B comparison 3, *p*<0.001). Similar results were obtained for path curvature (*F*_(5,234)_ = 18.1, *p*<0.00001); *IMPs* were significantly higher than control in matched-directions with VFB (Fig. 7C, comparison 2 *p*<0.001) but not without VFB (FFnv vs. FFnvR^−^, *p*>0.10). Reductions in curvature in the direction-matched groups were significantly higher with vision than without (Fig. 7C comparison 4, *p*<0.01). Note however that these subjects (FFnvR^−^) showed improvements similar to control force field without VFB (*p*>0.10) even though they continued making the same pattern of curved trajectories learned on day1.

When perturbation directions were non-matched, interference on the trajectory variables occurred both with and without vision (FFvR^+^ and FFnvR^+^). We found significantly lower-than-control *IMPs* for directional deviations (Fig. 7B comparisons 5–6 *p*<0.01) and path curvature (Fig. 7C comparisons 7–8 *p*<0.01).

In sum, adaptive changes in trajectory were facilitated during relearning of force fields with VFB when similar motor adjustments were required, but showed interference when motor adjustments conflicted. The fact that interference occurred both with and without vision suggests that it reflects the learning of opposite motor adjustments rather than mere differences in error feedback (proprioceptive vs. visual). Endpoint accuracy and variability remain unaffected by intervening adaptation to visuomotor rotation.

## Discussion

We report that when adapting to dynamic force perturbations, subjects achieved similar gains in terminal accuracy but generated different hand trajectories with and without visual feedback during movement. With continuous VFB, hand trajectories became straight but when visual information was limited to knowledge of success in achieving the target, trajectories remained curved over repeated blocks of learning. The differences in curvature suggest that proprioception may be used differently to achieve accuracy and precision when the visual feedback is only present at movement termination than during the entire movement.

Movement trajectories during later adaptation to visuomotor rotations reflected subjects' prior experience in force fields. Having earlier made curved trajectories in force fields without VFB, subjects kept curved trajectories even while they had to move the hand towards a different final position relative to the one used in force field. Intervening adaptation to visuomotor rotations influenced trajectories but not final position when relearning force fields the next day, consistent with differences in underlying control mechanisms. We suggest that the consistent curvature of trajectories learned initially with force fields represented a general strategy or the control policy used later to achieve accurate terminal control during rotation adaptation. Our results also indicate that prior learning history influences the choice and maintenance of control policy during adaptations.

### Curvature of hand trajectories

Previous studies of adaptation to dynamic perturbations that distort hand trajectories during reaching have stressed that with practice movements become both accurate and straight [1], [3]–[5] or slightly curved in the direction opposite to force field [28]. Computational models have suggested that common performance errors are utilized to minimize both endpoint variability and deviations from rectilinearity [6], [7]. Our findings do not support this notion but are in accord with the substantial curvature found previously during adaptation to visuomotor rotations when driven by errors in final hand position alone [15], [29]. Also consistent with this, adaptive compensation for inertial errors without VFB has been reported to be achieved at the cost of directional accuracy and independent of adaptive compensation for directional biases [4]. Thus, endpoint accuracy and trajectory shape appear to be adjusted by different sources of error. Note however that unlike our findings, the curvature reported by Scheidt and Ghez (2007) was more variable and proposed to emerge from interactions between learned trajectory and positional plans.

Since the dynamic perturbations decayed with hand velocity at movement termination, reductions in systematic and variable errors could have been achieved simply by increasing stiffness during the terminal segment of the movement (as suggested in the model of Scheidt and Ghez, 2007). Allowing initial trajectory direction to remain incompletely corrected relative to a hypothetical straight line conforms to a “minimum intervention” principle [30] and would have a lower energetic cost than fully offsetting deviations. However, adaptive regulation of an intended final posture would not account by itself for the trajectories we observed. First, the changes in initial directional deviations (Fig. 4), which reflect feedforward commands rather than feedback corrections, indicate that subjects did counteract the initial disturbance predictively. Second, the trial-to-trial variability in curvature was rapidly reduced even as terminal accuracy improved and then maintained around the same value across days. This suggests that trajectory curvature was planned and maintained at a desired value. Our finding that subjects generated trajectories with the same curvature when they later adapted to a visuomotor rotation supports this. We suggest that subjects adjusted their movement termination by rotating the learned but curved trajectory and the state estimates necessary to achieve accurate termination at the new location.

The transfer of a previously learned trajectory shape to a new task seen here is analogous to that reported by [31] that subjects continued to implement specific curvatures learned to avoid a seen obstacle even after the obstacle is removed.

### Roles of vision and proprioception in adaptive control of trajectory and final position

We suggest that the distinctive trajectories made with and without vision result from differences in the information conveyed by vision and proprioception. Visual feedback indicates the location of the hand relative to the target in extrinsic space. This allows hand location to be estimated in the same coordinates as the goal and errors to be computed without coordinate transformations. By contrast, proprioception conveys information about joint rotations and muscle lengths in intrinsic coordinates, which are unrelated to the location of visual targets without suitable calibration. Spatial estimates of hand position and movement direction determined through proprioception are also less precise than with vision [32] and drift substantially over time [33], [34].

The observations of Lackner and coworkers [2] demonstrate that the deviations from rectilinearity produced initially by Coriolis forces in a rotating room can be detected and corrected adaptively with proprioception alone. However, when guided by proprioception alone (i.e. in the dark) systematic endpoint deviations persisted. These errors were reduced (but were not eliminated) when subjects were allowed to touch a horizontal surface overlying the target. Thus, haptic feedback provided by contact forces on the finger could be used to correct terminal errors partially. The findings of Lackner and coworkers differ from ours in two respects. First, residual bias errors persisted with haptic feedback (variable errors were not measured) but were eliminated here. The failure to correct such errors completely seems attributable to the fact that subjects in the rotating room were not informed of the success or failure of successive responses. They therefore could only rely on their recall of the appropriate feedback in adjusting successive movements, and such recall can be assumed to degrade over time. Second, without VFB, our subjects made curved trajectories rather than the straight ones made by subjects in the rotating room. We hypothesize that this difference arose because the change in target color informed our subjects of trial success at movement endpoint. This would have allowed them to calibrate both proprioceptive and visual information at the single location where movements were expected to terminate, driving adjustments in inverse and forward models and recovery of terminal accuracy in all conditions. When continuous VFB was also available during movement, subjects could also directly calibrate proprioceptive, and visuospatial information associated with deviations from intended rectilinearity (see also [35]). Without VFB, proprioceptive feedback (and haptic) feedback could only be correlated with the intended changes in joint torques and angles to calibrate representations in intrinsic but not extrinsic space. Since calibration of sensory feedback does not transfer across modalities [19], we speculate that this might serve to generate reproducible trajectories for precise termination represented in joint- rather than in visuospatial coordinates. This may explain why adaptation to force fields was facilitated by matched visuomotor rotations with VFB but not without VFB. With VFB facilitation of learning would result in subjects' ability to identify the source of the early visual errors produced by the rotation to extrinsic rather than intrinsic space. Without VFB, proprioceptive errors would only be calibrated at the terminal location.

Differences between our findings and those of Franklin et al. [36] also require comment. In that study, subjects recovered accuracy and rectilinear trajectories whether movements were made with or without VFB. Unlike here, subjects had continuous VFB of their hand from the time movement ended near the target until it was returned to the starting position for the next trial. This information is critical; Only when it was denied and visual feedback limited to movement endpoints did curved trajectories develop during adaptation to visuomotor rotations [15] as occurred here. We believe that otherwise subjects prioritize straightness over terminal accuracy [29] as in the experiments of Lackner and DiZio. The results here are, however, more difficult to reconcile with those of [37] where subjects adapting to curl fields recovered rectilinearity without VFB. Further experiments, separating effects of prior experience, multiple target directions, present in that study but not here, will be needed to determine the origin of the discrepancy.

In sum, our results suggest that the strategies used during motor adaptation depend on subjects' prior experience and on the calibration of sensory channels conveying state estimates for different tasks. The different strategies correspond to different control policies, through which errors on individual trials determine specific compensatory adjustments on the next. A possible neural basis for separate control processes for stable and dynamically changing states, i.e. posture and movement, have been observed in the differential load-related activity of primary motor cortical neurons in primates [38]. Modulation of neuronal activity in the motor cortices has also been shown during adaptation to visuomotor rotations [25], [39]–[41] and viscous force fields [42]–[44]. The behavioral findings here suggest that motor cortical cells may also be differentially modulated by adaptation depending on available feedback and knowledge from prior experience.

## Supporting Information

Figure S1 Mean movement endpoints of each of the subjects in force field without visual feedback (FFnv,left) and with (FFv,right). Each circle is a subject's mean final hand position across the early (1∶40) and late (181∶220) trials of both days. Bar plot of the mean endpoints across all subjects in each group during the early and late phases of day1 and day2 training. Vertical lines are ±1 standard error of the mean.
(0.55 MB PDF)
