# Supplementary material for: Differences in Context and Feedback Result in Different Trajectories and Adaptation Strategies in Reaching
Source: PLoS One. 2009 Jan 16;4(1):e4214. doi: 10.1371/journal.pone.0004214 (PMC2625444; doi:10.1371/journal.pone.0004214)
Supplement: Figure S1 — Mean movement endpoints of each of the subjects in force field without visual feedback (FFnv,left) and with (FFv,right). Each circle is a subject's mean final hand position across the early (1∶40) and late (181∶220) trials of both days. Bar plot of the mean endpoints across all subjects in each group during the early and late phases of day1 and day2 training. Vertical lines are ±1 standard error of the mean. (0.55 MB PDF) [file pone.0004214.s001.pdf]

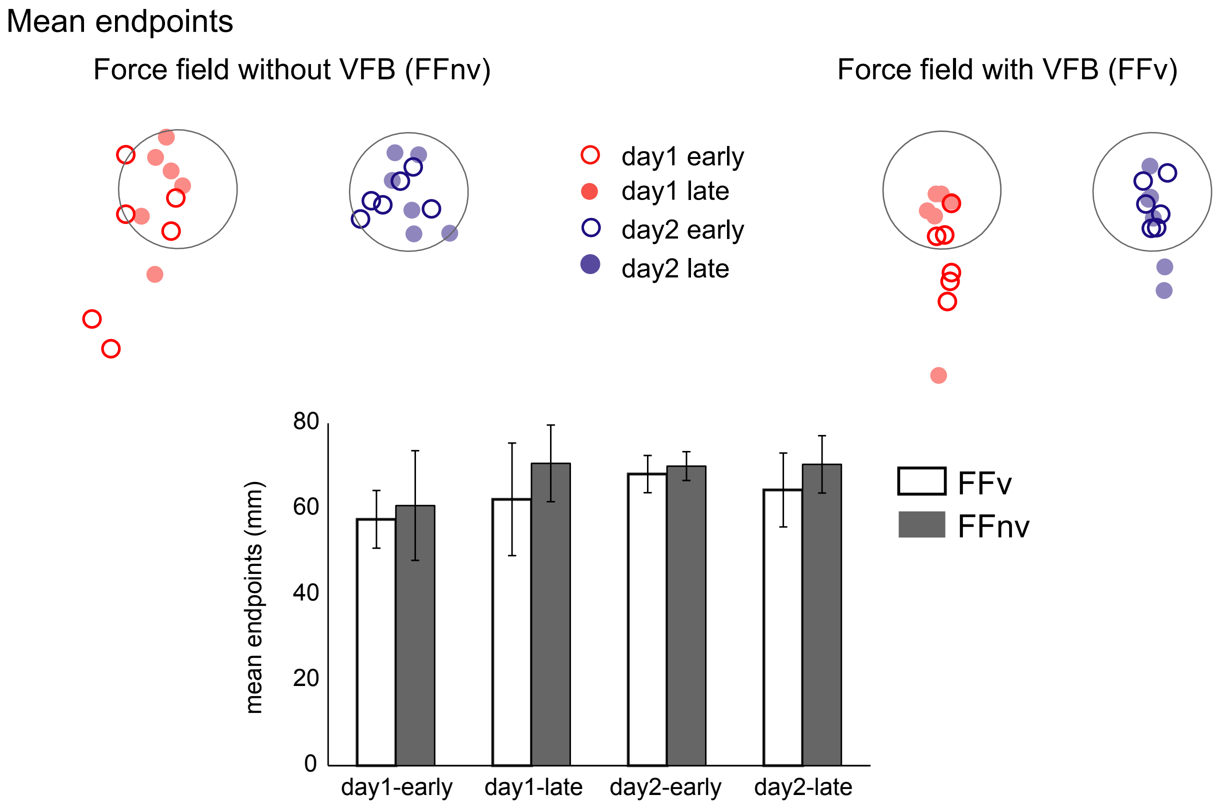

Figure S1. Mean movement endpoints of each of the subjects in force field without visual feedback (FFnv,left) and with (FFv,right). Each circle is a subject's mean final hand position across the early (1:40) and late (181:220) trials of both days. Bar plot of the mean endpoints across all subjects in each group during the early and late phases of day1 and day2 training. Vertical lines are ±1 standard error of the mean.
